# Markers of inflammation and immunological competence: Assessment in the early postoperative phase of cardiac surgery involving extracorporeal circulation

**DOI:** 10.5937/jomb0-56889

**Published:** 2025-11-05

**Authors:** Selma Mutevelić, Lejla Bajramović-Omeragić, Merima Šehić, Sumejja Baljević-Spahić, Kelle Belma Pehlivanović, Ermin Begović, Berina Hasanefendić, Ermina Mujičić, Marina Delić-Mašović, Drago Batinić

**Affiliations:** 1 Clinical Center University of Sarajevo, Department of Clinical Immunology, Sarajevo, Bosnia and Herzegovina; 2 County Hospital Zenica, Department of Medical Biochemistry and Immunology Diagnostics, Zenica, Bosnia and Herzegovina; 3 Clinical Center University of Sarajevo, Department of Clinical Pathology, Cytology and Human Genetics, Sarajevo, Bosnia and Herzegovina; 4 University of Sarajevo - Faculty of Pharmacy, Department of Pharmacology and Clinical Pharmacy, Sarajevo, Bosnia and Herzegovina; 5 Clinical Centre University of Sarajevo, Department of Clinical Biochemistry, Sarajevo, Bosnia and Herzegovina; 6 Clinical Centre University of Sarajevo, Clinic of Cardiovascular Surgery, Sarajevo, Bosnia and Herzegovina; 7 Oslo University Hospital Ulleval, Clinic for Laboratory Medicine, Immunology and Transfusion Medicine, Oslo, Norway; 8 University of Zagreb, School of Medicine, Zagreb, Croatia

**Keywords:** extracorporeal circulation, C-reactive protein, serum amyloid A, monocytes, ekstrakorporalna cirkulacija, C-reaktivni protein, serumski amiloid A, monociti

## Abstract

**Background:**

To gain insight into the role and relevance of inflammatory and immunological markers in the comprehensive assessment of a patient's immune response to surgical procedures. This study focused on investigating preoperative and postoperative serum levels dynamics of SAA, CRP and proportion of HLA-DR CD14 monocytes, CD14 monocytes, and pro-inflammatory monocytes CD16 T CD14 T in patients who underwent heart surgery using extracorporeal circulation (on-pump).

**Methods:**

An observational, prospective study was conducted at the Heart Center of the Clinical Center of the University of Sarajevo on 53 patients divided into 3 age groups: 50-59, 60-69, and 70-80. The serum levels of CRP and SAA were quantitatively determined by immunonephelometry. At the same time, flow cytometry technology was applied to measure the proportion of CD14 monocytes, HLA-DR CD14 monocytes, and pro-inflammatory CD16 CD14 monocytes.

**Results:**

Measured values of CRP; SAA, proportion of monocytes CD14, and proportion of pro-inflammatory monocytes CD16 CD14 are significantly increased postoperatively compared to the preoperative values (p &lt; 0.05). The proportion of HLA-DR CD14 monocytes is lower postoperatively compared to preoperative values (p &lt; 0.001). Furthermore, there are no significant gender differences in the preoperative or postoperative parameters (p &gt; 0.05), with the notable exception of the preoperative proportion of CD14 monocytes (p &lt; 0.05). The analysis of age-related differences indicates no significant changes in the observed preoperative and postoperative parameters among the defined age groups (p &gt;0.05).

**Conclusions:**

Early monitoring of inflammatory and immunological markers in the postoperative phase could be valuable for healthcare professionals to implement prompt interventions to mitigate negative outcomes.

## Abstract (sr)

**Uvod:**

Da bismo stekli uvid u ulogu i važnost upalnih i imunoloških markera u sveobuhvatnoj proceni imunološkog odgovora pacijenta na hirurški zahvat, naša studija se fokusirala na istraživanje preoperativne i postoperativne dinamike serumskih nivoa SAA, CRP i udela HLA-DR CD14 monocita, CD14 monocita, i proinflamatornih monocita CD16 CD14 kod pacijenata koji su bili podvrgnuti operaciji srca uz pomoć ekstrakorporalne cirkulacije (on-pump).

**Metode:**

U Centru za srce Kliničkog centra Univerziteta u Sarajevu sprovedena je opservacijska, prospektivna studija na 53 pacijenta podeljena u 3 starosne grupe: 50-59, 60-69, i 70-80. Nivoi CRP i SAA u serumu su kvantitativno određeni imunonefelometrijom. Istovremeno je primenjena tehnologija protočne citometrije za merenje udela HLA-DR CD14 monocita, CD14 monocita, i proinflamatornih CD16 CD14 monocita.

**Rezultati:**

Izmerene vrednosti CRP; SAA, udeo monocita CD14, i udeo proinflamatornih monocita CD16 CD14 su značajno postoperativno povećane u odnosu na preoperativne vrednosti (p &lt; 0,05). Udeo HLA-DR CD14 monocita je niži posle operacije u odnosu na preoperativne vrednosti (p &lt; 0,001). Nadalje, nema značajnih polnih razlika u preoperativnim ili postoperativnim parametrima (p &gt; 0,05), sa izuzetkom preoperativnog udela monocita (CD14) (p &lt; 0,05). Analiza starosnih razlika ukazuje da među definisanim starosnim grupama nije bilo značajnih promena u posmatranim preoperativnim i postoperativnim parametrima (p &gt; 0,05).

**Zaključak:**

Rano praćenje upalnih i imunoloških markera u postoperativnoj fazi bi moglo da bude od koristi za zdravstvene radnike za sprovođenje brzih intervencija usmerenih na ublažavanje negativnih ishoda.

## Introduction

The well-known cross-connection between the cardiovascular and immune systems, established through cytokines, hormones, and neurotransmitters, can be disrupted by numerous factors, resulting in inflammation, dysfunction, and tissue damage [1]. The development of an inflammation process following cardiac surgery is a common response [2]. Extracorporeal circulation (ECC) is one of the most impactful advances in cardiac surgery. However, it negatively affects the host's immune system by eliciting a strong innate immune response, which is often expressed as systemic inflammatory response syndrome (SIRS) [3]. Recent research showed that systemic inflammatory response after cardiac surgery is associated with increased mortality [4]. The negative impact of ECC can be primarily linked to alterations in arterial blood flow caused by the exposure of blood to non-physiological surfaces and substances during the ECC interval. Such exposure initiates a cascade of reactions driven by cytokines, which activate the endothelium of blood vessels, resulting in further inflammatory response predominantly mediated by neutrophils [3] [5] [6]. In addition to a strong inflammatory response, patients after the EC also show signs of reduced general immune competence, e.g. decreased expression of monocyte human leukocyte antigen (HLA-DR) and cluster of differentiation (CD) 86 [6]. The aforementioned contribute to the classification of patients post-EC as a high-risk demographic for the emergence of infections and their potential repercussions.

Following cardiac surgery that employs ECC, immune parameters can act as preliminary indicators of potential inflammation or infection risk [6]. Increasing serum levels of immune parameters such as C-reactive protein (CRP), procalcitonin (PCT), interleukins (ILs), tumour necrosis factor-alpha (TNFα), white blood cell (WBC), and monocyte count commonly serve as risk predictors of an inflammatory process after the surgery [5] [6] [7]. Furthermore, serum amyloid A (SAA), a significantly conserved protein that functions as an acute phase reactant, is produced in response to both inflammation and infection and can be used to predict post-surgical outcomes [8] [9]. Evidence suggests that SAA is better than CRP as a postoperative inflammatory marker [10].

Therefore, this study aimed to determine and monitor preoperative and postoperative serum levels dynamics of SAA, CRP, proportion of HLA-DR^+^ CD14^+^ monocytes, CD14^+^ monocytes, and proinflammatory monocytes CD16^+^CD14^+^ in patients who underwent heart surgery using extracorporeal circulation (on-pump) to establish their role in extensive understanding of a patient's immune reaction to surgical procedures and the potential risk of com( plications, including infection, sepsis, or organ dysfunction.

## Materials and methods

### Patients and study design

An observational, prospective study was conducted at the Heart Center of the Clinical Center of the University of Sarajevo on patients who underwent heart surgery with a classic median sternotomy using extracorporeal circulation (on-pump). A total number of 53 patients (38 males and 15 females, mean age 64.7±7.46 years) were included in the study, and written informed consent was obtained from all participants. This study was conducted using the principles of the Declaration of Helsinki, and the research protocol was approved by the Institute for Research and Development of the Clinical Center University of Sarajevo (Approval number: 0207-34-198).

The total examined patient population (N=53) was divided into 3 age groups: 50-59 (N = 13; 24.5%), 60-69 (N=26; 49.1%), and 70-80 (N = 14, 26.4%). Patient information such as age, gender, and relevant medical history were collected from the hospital information system. During preoperative preparation, all patients underwent a standard diagnostic procedure and clinical and laboratory evaluation that ruled out the presence of infection in the patient. General obligatory criteria for surgery admission were the following: proper preoperative swabs of the nose, throat, and skin, leukocytes and CRP in reference levels, regulated glycemic value, negative markers for hepatitis B and C, negative markers for human immunodeficiency virus (HIV) infection as well as the absence of clinical and laboratory signs of infections. Criteria for including patients in the study were the following: (a) patients who signed the informed consent; (b) patients who undergo an elective surgical procedure of implanting bypasses on the blood vessels of the heart with the use of extracorporeal circulation (on-pump); (c) patients over the age of 40 and (d) patients whose extracorporeal circulation lasted less than 90 minutes up to 65 years. Criteria for excluding patients from the study were the following: (a) transplanted patients; (b) patients with verified HIV infection or other immunological deficiencies; (c) patients who were on corticosteroid therapy preoperatively; (d) patients with suspected preoperative infection and (e) patients with metastatic disease.

Extracorporeal circulation was performed with non-pulsatile roller pumps and a membrane oxygenator (Affinity, Avecor, Bellshill) in mild hypothermia (3234°C) with a flow rate of 2.4 to 3.0 L/min/m^2^. An anesthesiologic technique for cardiac surgery was performed according to standard general endotracheal balanced intravenous and inhalation anaesthesia.

### Determination of markers of inflammation and immunological competence

The following markers were determined: serum amyloid A (SAA), C-reactive protein (CRP), the proportion of CD14^+^ monocytes from the CD45^+^ leukocytes, and the proportion of HLA-DR^+^CD14^+^ monocytes and pro-inflammatory CD16^+^CD14^+^ monocytes from the CD14^+^ monocytes.

The serum levels of CRP (mg/L) and SAA (mg/L) were quantitatively determined by immuno-nephelometry using an N-Latex SAA and CRP reagents on Nephelometer BNII (Siemens Healthcare Diagnostics Products GmBH, Marburg, Germany) according to the manufacturer's protocol. Flow cytometry technology was applied to measure the proportion of HLA-DR^+^CD14^+^ monocytes (%) and the proportion of pro-inflammatory CD16^+^CD14^+^ and CD14^+^ monocytes (%) on a bD FACSCanto™ II Clinical Flow Cytometry System. CD-markers' expressions on monocytes were determined by a panel of CD16/HLA-DR/CD45/CD14 monoclonal antibodies. Monoclonal antibodies were obtained from Becton Dickinson (San Jose, CA, USA). After processing 10,000 cells, the acquisition was stopped, and data analysis focused on the percentage of cells positive for certain surface markers. The blood samples for determination of immunological markers of inflammation and competence were obtained from the patients preoperatively (PreOP) and on the 2^nd^ day after the surgery (PostOP). Preoperative collection of the blood samples was taken just before the patient's induction of general anaesthesia in the operating room. Postoperative collection of blood samples was taken 24 hours after the surgery procedure was completed. They were collected into two test tubes: (1) a tube with incorporated gel for determination of serum CRP and SAA levels and (2) a tube with EDTA for determination of the proportion of HLA-DR^+^ CD14^+^ monocytes and the proportion of CD14^+^ and pro-inflammatory CD16^+^CD14^+^ monocytes. The blood samples for tube 1 were collected from the peripheral intravenous pathway and centrifuged at 4000 rpm for 5 min, while samples for tube 2 were collected from a three-lumen central venous catheter (Arrow multi-lumen Central Venous Catheter 7 Fr.20 cm) and proceeded further preparations for analysis on flow cytometry, which further preparational steps were undertaken: total 5 μL BD single antibodies (BD™ FITC Mouse Anti-Human CD16, BD™ PE Mouse Anti-Human HLA-DR, BD™ PerCP Mouse Anti-Human CD45, BD™ CD14 APC) were added to the bottom of the marked test tube and vortexed with 100 microliters peripheral blood samples and incubated in the dark for 15 min. at room temperature. After the incubation, samples were vortexed with 2 mL of FACS Lysing solution (BD Biosciences, San Jose, USA) and again incubated in the dark for 10 minutes at room temperature. Following centrifugation for 4 minutes at 2200 revolutions per minute, the supernatant was poured off afterwards and then vortexed with 2 mL of Cell WASH solution (BD Biosciences, San Jose, USA). Afterwards, samples were centrifuged for 4 minutes at 2200 revolutions per minute, and the supernatant was poured, vortexed, and then analyzed within the next 24 hours at the Department of Clinical Immunology.

### Statistical analysis

The Statistical Package for Social Sciences (IBM SPSS) program, version 23.0 (SPSS Inc, Chicago, Illinois, SAD), was used for statistical analysis. Mean, standard deviation, median, interquartile range (IQR), and frequency values were used for the descriptive statistics of the data. Data distribution was tested using the Kolmogorov-Smirnov and Shapiro-Wilk tests. Mann-Whitney U test was used to compare genders, Kruskal Wallis was used to compare age groups, and the Wilcoxon Signed Rank test was used for repeated measurements (preoperative and postoperative). Pearson correlation analysis was used to assess the relationships between the parameters. Accepted statistical significance was at the level of p<0.05.

## Results

Table 1 shows the median values of all the observed parameters, which are significantly different in terms of the period of measurement (preoperative vs. postoperative). Measured values of CRP (mg/L), SAA (mg/L), proportion (%) of CD14^+^ monocytes, and proportion of pro-inflammatory monocytes CD16^+^CD14^+^ (%) are significantly increased postoperatively compared to the preoperative values (p<0.05). The proportion of HLA-DR^+^CD14^+^ monocytes is lower postoperatively compared to preoperative values (p<0.001) (Table 1).

**Table 1:** Measurement and comparison of investigated parameters preoperatively and postoperatively. Values represent medians (lower - upper quartile), p - significance between groups, N - number of patients, PreOP - preoperative values; PostOP - postoperative values; CRP - C reactive protein; SAA - serum amyloid A; CD14+ - monocyte; HLADR+CD14+ - activated monocyte; CD16+CD14+ - pro-inflammatory monocyte.

Parameter	PreOP period<br>(N = 53)	PostOP period<br>(N = 53)	Z value	p-value
CRP (mg/L)	0.01 (0.01-0.010.)	120.00 (79.10-156.50)	-6.325	<0.001
SAA (mg/L)	7.20 (5.00-11.25)	485.00 (165.00-1155.00)	-5.998	<0.001
CD14^+^ (%)	6.40 (5.05-7.85)	7.70 (5.45-9.75)	-2.692	0.007
HLA DR+CD14^+^ (%)	41.20 (30.55-60.30)	20.50 (13.65-30.25)	-5.830	<0.001
CD16+CD14^+^ (%)	1.10 (0.45-2.30)	3.60 (2.25-8.10)	-5.467	<0.001

The results of the study indicate that there are no significant gender differences in the measured preoperative or postoperative parameters (p>0.05), with the notable exception of the preoperative proportion of CD14^+^ monocytes (p<0.05), as shown in Table 2. Specifically, male patients demonstrated a significantly higher preoperative proportion of CD14^+^ monocytes than female patients; however, this difference was not present in the postoperative assessment. Additionally, the study found no significant age differences between male and female patients.

**Table 2:** Gender differences of investigated parameters preoperatively and postoperatively. IQR - interquartile range, p - significance between groups, N - number of patients, PreOP - preoperative values; PostOP - postoperative values; CRP - C reactive protein; SAA - serum amyloid A; CD14^+^ - monocyte; HLADR^+^CD14^+^ - activated monocyte; CD16^+^CD14^+^ - pro-inflammatory monocyte.

Period	Parameter	Gender	N	Median	IQR		Mann-Whitney<br>U	p-value
					25%	75%		
PreOP	Age (years)	Male	38	63.50	58.50	70.25	225.50	0.239
		Female	15	67.00	62.00	73.00		
	CRP (mg/L)	Male	38	0.01	0.01	0.01	279.00	0.862
		Female	15	0.01	0.01	0.01		
	SAA (mg/L)	Male	38	7.00	4.40	10.52	231.50	0.291
		Female	15	9.00	5.00	16.80		
	CD14^+^ (%)	Male	38	6.60	5.37	8.05	163.50	0.016
		Female	15	5.10	4.40	7.20		
	HLADR^+^CD14^+^<br>(%)	Male	38	44.75	35.82	61.35	204.00	0.110
		Female	15	31.30	29.70	45.10		
	CD16^+^CD14^+^<br>(%)	Male	38	1.10	0.57	2.22	251.50	0.507
		Female	15	0.60	0.30	2.40		
PostOP	CRP (mg/L)	Male	38	126.00	71.27	153.25	284.00	0.984
		Female	15	113.00	83.00	168.00		
	SAA (mg/L)	Male	38	469.50	157.25	1260.00	275.50	0.851
		Female	15	597.00	321.00	750.00		
	CD14^+^ (%)	Male	38	7.70	5.67	9.05	273.00	0.813
		Female	15	6.70	4.90	10.30		
	HLADR^+^CD14^+^<br>(%)	Male	38	21.55	14.47	30.12	216.00	0.173
		Female	15	17.10	5.10	31.10		
	CD16^+^CD14^+^<br>(%)	Male	38	3.75	2.20	7.37	282.00	0.953
		Female	15	3.40	2.30	9.50		

Furthermore, the results of age-related differences indicate that there were no significant changes in the observed preoperative and postoperative parameters among the defined age groups (p>0.05) (Table 3). Therefore, there was no need to proceed with post hoc analysis.

**Table 3:** Age differences of investigated parameters preoperatively and postoperatively. IQR - interquartile range, p - significance between groups, N - number of patients, PreOP - preoperative values; PostOP - postoperative values; CRP - C reactive protein; SAA - serum amyloid A; CD14+ - monocyte; HLADR+CD14+ - activated monocyte; CD16+CD14 + - pro-inflammatory monocyte.

Period	Parameter	Age group	N	Median	IQR		Chi-Square	p-value
					25%	75%		
PreOP	CRP (mg/L)	50-59	13	0.01	0.01	0.01	1.19	0.550
		60-69	26	0.01	0.01	1.06		
		70-80	14	0.01	0.01	1.26		
	SAA (mg/L)	50-59	13	8.30	5.55	11.20	0.75	0.686
		60-69	26	6.35	4.82	12.45		
		70-80	14	7.25	4.47	11.80		
	CD14^+^ (%)	50-59	13	6.40	4.65	7.10	1.28	0.526
		60-69	26	6.45	5.27	7.92		
		70-80	14	6.60	4.72	9.67		
	HLADR^+^CD14^+^ (%)	50-59	13	40.10	35.55	53.30	3.1	0.210
		60-69	26	45.50	31.12	66.30		
		70-80	14	34.60	21.97	52.37		
	CD16^+^CD14^+^(%)	50-59	13	0.90	0.45	2.35	0.24	0.885
		60-69	26	1.20	0.47	2.12		
		70-80	14	0.90	0.37	2.42		
PostOP	CRP (mg/L)	50-59	13	119.00	86.60	166.50	0.02	0.987
		60-69	26	128.00	74.32	155.25		
		70-80	14	116.50	86.27	171.75		
	SAA (mg/L)	50-59	13	250.00	164.00	696.50	1.53	0.465
		60-69	26	512.00	158.75	1207.50		
		70-80	14	640.00	183.75	1407.50		
	CD14^+^ (%)	50-59	13	6.20	5.20	8.20	1.67	0.433
		60-69	26	7.70	5.17	10.47		
		70-80	14	7.75	5.75	9.77		
	HLADR^+^CD14^+^ (%)	50-59	13	20.50	14.05	31.20	0.23	0.889
		60-69	26	23.00	12.17	31.02		
		70-80	14	19.10	13.42	29.45		
	CD16^+^CD14^+^(%)	50-59	13	4.1	2.40	9.10	0.60	0.738
		60-69	26	3.30	1.77	7.85		
		70-80	14	3.05	2.50	9.35		

Table 4 shows a preoperative correlation analysis between investigated parameters. Results indicate that the age of the patients did not show a significant correlation with any of the examined preoperative parameters. Preoperative CRP and SAA showed a statistically significant positive correlation (p<0.05). Likewise, preoperative CRP showed a statistically significant positive correlation (p=0.032) with preoperative pro-inflammatory monocytes (CD16^+^CD14^+^). Preoperative pro-inflammatory monocytes (CD16^+^CD14^+^) showed a statistically significant negative correlation with preoperative total CD14^+^ monocytes (p=0.018) and a positive correlation with preoperative HLA-DR^+^CD14^+^monocytes (p=0.003).

**Table 4:** Preoperative correlation analysis between investigated parameters. p - significance between groups, PreOP - preoperative values; PostOP - postoperative values; CRP- C reactive protein; SAA - serum amyloid; ACD14+ - monocyte; HLADR+CD14+ - activated monocyte; CD16+CD14+ - proinflammatory monocyte.

PreOP parameters		Age (years)	CRP (mg/L)	SAA (mg/L)	CD14^+^ (%)	HLADR^+^CD14^+^
CRP (mg/L)	Pearson r	0.059				
	p-value	0.673				
SAA (mg/L)	Pearson r	-0.011	0.690			
	p-value	0.938	<0.001			
CD14^+^ (%)	Pearson r	0.188	-0.195	-0.091		
	p-value	0.177	0.161	0.516		
HLADR^+^CD14^+^<br>(%)	Pearson r	-0.112	-0.051	-0.213	-0.156	
	p-value	0.426	0.715	0.126	0.264	
CD16^+^CD14^+<br>^(%)	Pearson r	0.131	0.295	0.142	-0.324	0.401
	p-value	0.350	0.032	0.312	0.018	0.003

The findings from the postoperative correlation analysis of the examined parameters are summarized in Table 5. The analysis indicates that patient age did not exhibit a statistically significant correlation with any of the postoperative parameters assessed. Notably, postoperative C-reactive protein (CRP) demonstrated a statistically significant negative correlation (p = 0.026) with total CD14^+^ monocytes. However, postoperative pro-inflammatory monocytes (CD16^+^CD14^+^) did not reveal significant correlations with either total CD14^+^ monocytes or HLA-DR^+^CD14^+^ monocytes, which is consistent with the preoperative findings. Additionally, the postoperative serum amyloid A (SAA) level did not show any significant correlations with the parameters evaluated.

**Table 5:** Postoperative correlation analysis between investigated parameters. p - significance between groups, PreOP - preoperative values; PostOP - postoperative values; CRP - C reactive protein; SAA - serum amyloid A; CD14+ - monocyte; HLADR+CD14+ - activated monocyte; CD16+CD14+ - proinflammatory monocyte.

PostOP parameters		Age (years)	CRP (mg/L)	SAA (mg/L)	CD14^+^ (%)	HLADR^+^CD14^+^ (%)
CRP (mg/L)	Pearson r	-0.036				
	p-value	0.797				
SAA (mg/L)	Pearson r	0.095	0.234			
	p-value	0.497	0.092			
CD14^+^ (%)	Pearson r	0.172	-0.305	-0.102		
	p-value	0.218	0.026	0.465		
HLADR^+^CD14^+^ (%)	Pearson r	-0.084	-0.217	0.094	-0.166	
	p-value	0.549	0.118	0.503	0.235	
CD16^+^CD14^+^ (%)	Pearson r	-0.089	0.025	-0.165	0.076	0.157
	p-value	0.524	0.860	0.239	0.591	0.262

## Discussion

To gain insight into the role and relevance of inflammatory and immunological markers in the comprehensive assessment of a patient's immune response to cardiac surgical procedure, our study focused on investigating preoperative and postoperative serum levels dynamics of SAA, CRP proportion of CD14^+^ monocytes and proportion of HLA-DR^+^ CD14^+^ and pro-inflammatory monocytes CD14^+ ^CD16^+^ in patients who underwent cardiac surgery using extracorporeal circulation (on-pump).

The obtained findings confirm the development of the acute inflammatory response syndrome triggered by ECC during cardiac surgery due to a significant postoperative increase in CRP, SAA, monocyte populations CD14^+^ and pro-inflammatory CD16^+^ CD14^+^ monocytes and reduced proportion of HLA-DR^+^CD14^+^ monocytes. Postoperative increase in serum levels of CRP and SAA was expected as their elevation is a well-established marker of postoperative inflammation and infection [11]. In some cases, the elevation of SAA may be more pronounced and quicker than CRP, though both are often elevated together [12]. This is consistent with our results, as our values of CRP showed a statistically significant positive correlation with SAA (p<0.05) as well as with pro-inflammatory monocytes (CD16^+^CD14^+^). The most investigated biomarkers of postoperative cardiac sepsis in terms of their diagnostic value are CRP, procalcitonin, SAA, and IFN-g induced protein 10 (interferon-gamma). However, the ideal biomarker has not yet been identified. Efforts are made towards developing multiple point-of-care (POC) systems for measuring biomarkers of sepsis [13]. Haining et al. [14] reported that SAA proved to be a better marker of neonatal sepsis than CRP However, it is noted that the combined measurement of SAA, CRP, and PCT (procalcitonin) will have a more significant impact on the sepsis diagnosis [14].

In agreement with our findings, it has been shown that patients undergoing ECC during cardiac surgery reveal evidence of reduced overall immune competence, which is mainly manifested through a decrease in levels of HLA-DR^+^CD14^+^ monocytes, amongst other parameters such as CD86 and TNF-α [3]. Several investigators have identified a correlation between the extent of immunosuppression following surgery, primarily the reduction in H LA-DR expression, and the later onset of postoperative infections or complications in both general and cardinal surgery [15] [16] [17]. Also, it has been shown that reduced HLA- DR expression on CD14+ monocytes is more pronounced and long-lasting in patients who develop sepsis and even more pronounced in those patients who eventually die [18]. Lekkou et al. reported that the expression level of HLA-DR^+^CD14^+^ monocyte alongside the serum level of IL-10 was the best predictor of the development of infection in patients after ECC on the first postoperative day [19].

Monocytic CD14 serves as a key protein involved in the recognition of pathogens and the activation of immune responses [20]. Consistent with our results, Sharygin et al. highlighted a notable rise in CD14^+^ monocytes after surgical procedures, which is indicative of the body's innate immune response aimed at managing stress, tissue damage, and possible microbial infections developed during the recovery phase [21]. Currently, there is an increasing focus on the significance of the CD14 protein in the pathogenesis of non-infectious conditions, including autoimmune disorders, metabolic syndromes, and cardiovascular diseases [20] [22]. Furthermore, the tendency of proinflammatory CD14^+^CD16^+^ monocytes to adhere to artificial membranes during ECC has been previously reported [23]. This adhesion is critical as it results in the activation of monocytes and the release of cytokines that contribute to the inflammatory syndrome. The aforementioned are consistent with our findings, which revealed a significant postoperative rise in pro-inflammatory CD16^+^CD14^+^ monocytes. According to Rogacev et al. [24], monitoring the dynamics of these monocytes is essential as it serves as a significant predictor for cardiovascular risk.

Furthermore, our results revealed that there are no significant differences when considering gender and age in inflammatory responses based on values of SAA, CRP, the proportion of HLA-DR^+^CD14^+^ monocytes, and the proportion of CD14^+^ monocytes and pro-inflammatory monocytes CD14^+^CD16^+^ in patients who underwent heart surgery using extracorporeal circulation. This suggests that the inflammatory response following this type of cardiac surgery is relatively homogenous across these demographic categories. As a result, this insight can inform the creation of generalized postoperative care protocols that do not necessitate considerable adjustments based on age and gender.

## Conclusion

Major surgical interventions such as cardiac surgery have been shown to have profound effects on the immune system. It is of great clinical importance to understand a patient's immune response after undergoing a surgical procedure, as well as to identify potential biomarkers for monitoring and managing postoperative inflammation and infections. Findings from our study provide valuable insight into the relationship between markers of general immune competence: the proportion of CD14^+^ monocytes, the proportion of HLA-DR^+^CD14^+^ monocytes, and markers of inflammation: the proportion of pro-inflammatory monocytes CD16^+^14^+^, and dynamics of CRP and SAA levels in patients who underwent cardiac surgery using ECC. These markers could be useful tools in the follow-up of patients who underwent cardiac surgery using ECC to monitor the inflammatory response postoperatively. Therefore, they can be considered possible predictors of potential complications such as infection and other conditions related to immunocompromised reactions.

### Limitations

Our study is limited by the relatively small sample size and the fact that only one postoperative time point was evaluated. Assessing additional postoperative time points would provide more robust evidence to validate our findings. For future studies, it would be beneficial to involve a larger sample of patients and to implement a broader range of postoperative measurement intervals. Also, it would be useful to determine more immunological markers, markers of inflammation such as Il-6 or TNF-α, as well as to make correlations with clinical outcomes after surgery.

## Dodatak

### List of abbreviations

ECC, extracorporeal circulation;<br>SIRS, systemic inflammatory response syndrome;<br>HLD DR, human leukocyte antigen;<br>CD, a cluster of differentiation;<br>CRP C-reactive protein;<br>PCT, procalcitonin;<br>Ils, interleukins;<br>TNFa, tumour necrosis factor-alpha;<br>WBC, white blood cell;<br>SAA, serum amyloid;<br>HIV, human immunodeficiency virus;<br>EDTA, ethylenediaminetetraacetic acid;<br>SPSS, statistical package for social sciences.

### Conflict of interest statement

All the authors declare that they have no conflict of interest in this work.
